# Upper-extremity musculoskeletal disorders: how many cases can be prevented? Estimates from the COSALI cohort

**DOI:** 10.5271/sjweh.3911

**Published:** 2020-10-30

**Authors:** Aboubakari Nambiema, Julie Bodin, Natacha Fouquet, Sandrine Bertrais, Susan Stock, Agnès Aublet-Cuvelier, Alexis Descatha, Bradley Evanoff, Yves Roquelaure

**Affiliations:** 1 Univ Angers, CHU Angers, Univ Rennes, Inserm, EHESP, Irset (Institut de recherche en santé, environnement et travail) - UMR_S 1085, Angers, France; 2 Santé publique France, the French national public health agency, Direction of Occupational Health, EpiprevTMS team associated with the ­University of Angers, Angers, France; 3 INSPQ - Institut National de Santé Publique du Québec, Montréal, QC, Canada; 4 Department of Social & Preventive Medicine, University of Montréal, QC, Canada; 5 INRS, Département Homme au travail, Vandœuvre, France; 6 Inserm, UMS 011, unité cohortes épidémiologiques en population, Villejuif, France; 7 Division of General Medical Sciences, Washington University School of Medicine, St. Louis, MO, USA, Saint-Louis, USA

**Keywords:** cohort study, France, MSD, musculoskeletal disease, occupational risk factor, physical exertion, preventable case, prevention

## Abstract

**Objective:**

This study aimed to estimate the proportion and number of incident upper-extremity musculoskeletal disorders (UEMSD) cases attributable to occupational risk factors in a working population.

**Methods:**

Between 2002−2005, occupational physicians randomly selected 3710 workers, aged 20–59, from the Pays de la Loire (PdL) region. All participants underwent a standardized clinical examination. Between 2007−2010, 1611 workers were re-examined. This study included 1246 workers who were free of six main clinically diagnosed UEMSD at baseline but were diagnosed with at least one of these UEMSD at follow-up [59% of men, mean age: 38 (standard deviation 8.6) years]. Relative risks and population-attributable fractions (PAF) were calculated using Cox multivariable models with equal follow-up time and robust variance. The total number of incident UEMSD in the PdL region was estimated after adjustment of the sample weights using 2007 census data. The estimated number of potentially avoidable UEMSD was calculated by multiplying PAF by the total number of incident UEMSD in PdL.

**Results:**

At follow-up, 139 new cases of UEMSD (11% of the study sample) were diagnosed. This represented an estimated 129 320 incident cases in the PdL in 2007. Following adjustment for personal factors, 26 381 (20.4% of all incident UEMSD) were attributable to high physical exertion, 16 682 (12.9%) to low social support, and 8535 (6.6%) to working with arms above shoulder level.

**Conclusions:**

A large number and important proportion of incident UEMSD may be preventable by reducing work exposures to physical exertion and working with arms above shoulder level as well as improving social support from co-workers/supervisors.

Upper-extremity musculoskeletal disorders (UEMSD) are among the leading causes of morbidity and work disability in the working population of industrialized and developing countries (1, 2). Today, these disorders are a major concern for occupational and public health due to the considerable human, social and occupational costs (2–4). According to Eurostat, MSD account for almost 60% of work-related problems and are, therefore, the main work-related disease in the European Union (5). In France, according to 2018 social health insurance data, UEMSD accounted for 80% (39 555 cases) of all occupational diseases (6).

Numerous epidemiologic studies in working populations have identified a wide range of personal and work-related risk factors associated with UEMSD (7–12). While some personal attributes (eg, age) cannot be modified by preventive or medical interventions, exposure to work-related factors can potentially be modified by workplace-based interventions (13–15). In order to target and prioritize risk factors for more effective interventions in the workplace, it would be useful to quantify the proportion and number of UEMSD cases that could be prevented if exposure to these factors were reduced to levels that minimize the risk of UEMSD. Such information may provide an estimate of the theoretically maximum potential impact of preventive programs in the workplace (16). Identifying the occupational risk factors of UEMSD with the greatest impact may help public health practitioners and policy-makers prioritize interventions that reduce exposure to these factors (17).

At the population level, the effect of a risk factor on a disease can be quantified by the computation of the population attributable fraction (PAF) by taking into account both the strength of the association between a risk factor and a disease and the prevalence of that risk factor within the population (18). Thus, the PAF provides an estimate of the proportion of cases that would not have occurred if the exposure to a risk factor was reduced or eliminated (19); and it is therefore relevant to decision-making in public health.

Although there is extensive literature providing evidence of the associations between UEMSD and exposures in the workplace, few studies have assessed the PAF in the general working population (20–25) and specifically exposed populations (26–28). Moreover, none of these studies has estimated the number of incident UEMSD cases attributable to occupational risk factors. Identifying potential modifiable risk factors that preventive interventions could target to avert the greatest number of cases would improve the prevention of UEMSD in the working population. Consequently, the objective of this study was to estimate, using the multivariable model we previously obtained (29), the proportion and number of incident UEMSD cases attributable to occupational risk factors in the working population of the French region of Pays de la Loire (PdL).

## Methods

### Study population

We used data from the COSALI cohort, a prospective study of MSD and their risk factors in the working population based on two successive surveys of workers from the PdL region (30, 31). The region accounts for about 6% of the French working population and its diversified socioeconomic structure is similar to that of France as a whole (30).

Between 2002–2005, 83 occupational physicians (OP) (18% of OP in the region) volunteered to take part in the study. They selected 3710 workers (2161 men, 1549 women) at random (out of 184 600 under the surveillance of the 83 OP, 2.0%). More than 90% of the selected workers participated in this study (<10%: no shows, refusals, duplications). Women were slightly underrepresented in the sample (42% versus 47% in the region, P<0.001). Overall, the distribution of occupations in the sample was close to that of the regional workforce, except for the occupations not surveyed by OP (eg, farmers, shopkeepers, and self-employed workers). Data on personal characteristics and working conditions were collected by a self-administered questionnaire. The OP conducted a clinical examination of the participants using a standardized clinical protocol that strictly applied the methodology and clinical tests of the European consensus criteria to diagnose work-related UEMSD (WRUEMSD) (32). Each participating OP in charge of medical surveillance of salaried workers received guidelines describing the clinical protocol (including diagnostic criteria charts and photographs of clinical tests) and underwent a 3-hour training program to standardize clinical examinations. Between 2007–2010, the OP re-examined 1611 workers using the same procedure as the initial assessment [see (30, 31) for more details about the COSALI cohort].

This study received approval from France’s Advisory Committee on the Processing of Information in Health Research (“CCTIRS”) and the National Committee for Data Protection (“CNIL”), initially in 2001 and again in 2006. Each worker provided written informed consent prior to participation.

For the present study, 1228 of the workers included at baseline did not participate in the follow-up due to death, retirement, parental leave, long-term sick leave, unemployment, etc. Of the remaining 2482 participants, 23 refused to participate and 848 workers did not undergo the second clinical examination because they had no mandatory examination scheduled during the follow-up period. A comparison of baseline characteristics of workers who attended a follow-up (ie, second clinical examination) and workers who did not attend was described previously (29) and demonstrated that workers who did not attend a follow-up were significantly more likely to be younger, temporary workers or individuals with a short length of service at baseline.

**Figure 1:**
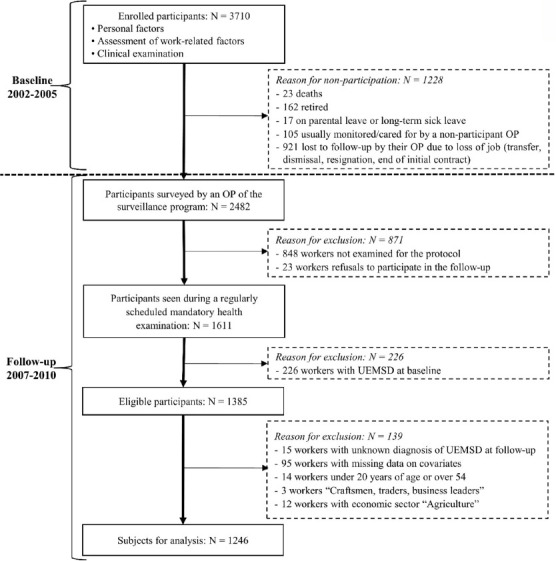
Participant flow diagram.

Among the 1611 participants with a standardized clinical examination at follow-up, 226 had at least one UEMSD at baseline and were excluded from the present study. Out of the 1385 eligible participants, ie, free of UEMSD at baseline, 110 workers with missing data for exposure or UEMSD were excluded (figure 1). In addition, 29 workers were excluded in order to standardize the auxiliary variables data between the sample and the external source, ie, the 2007 French population census data of the PdL region, before applying the weighting method (the calibration approach). After exclusions among eligible participants, the final study sample for current analyses consisted of 1246 participants.

### Outcome definition

Incident cases of UEMSD were defined as workers free of the six main clinically diagnosed UEMSD at baseline but who met the criteria for at least one of the disorders at follow-up. This definition was based on the European consensus criteria to diagnose WRUEMSD for health surveillance or epidemiologic studies (32). This consensus is intended to facilitate more consistent collection, recording and reporting of information on WRUEMSD across the European Union by providing evidence-based or consensus-based case definitions and criteria for their identification and categorization. The six main diagnosed UEMSD were: (i) rotator cuff syndrome, (ii) lateral epicondylar tendinopathy, (iii) carpal tunnel syndrome (CTS), (iv) ulnar tunnel syndrome, (v) flexor-extensor peritendinitis or tenosynovitis of the forearm-wrist region, and (vi) De Quervain’s tenosynovitis. Details regarding measurement of these disorders have been previously described (31).

### UEMSD risk factors

Only baseline factors retained as independent risk factors of UEMSD that were previously in the same sample (29) were assessed in this study.

*Personal factors* included sex, age divided into three categories (<35, 35–44 and ≥45 years) and overweight/obesity [body mass index (BMI) ≥25.0 kg/m^2^ (33)].

*Work-related biomechanical factors* [assessed using the European consensus criteria (32)] included: high repetitiveness of tasks (≥4 hours/day); repeated/sustained posture with arms above shoulder level (≥2 hours/day); repeated/sustained elbow movements (flexion/extension) (≥2 hours/day); and wrist twisting movements (≥2 hours/day). Concerning the exposure “repeated/sustained shoulder abduction”, workers who responded “rarely (<2 hours/day)”, “often (2–4 hours/day)” or “always (≥4 hours/day)” were defined as being at risk of this posture (30). The questionnaire presented awkward postures in picture form to facilitate workers’ understanding and thus increase the validity of posture self-assessment (34). The perceived physical exertion was evaluated using the Borg Rating Perceived Exertion (RPE) scale (35), ranging from 6 (no exertion at all) to 20 (maximal exertion). RPE was dichotomized using the threshold (Borg RPE scale ≥13) proposed by the French National Research and Safety Institute for the Prevention of Occupational Accidents and Diseases (INRS cut-offs) (36).

*Work-related psychosocial factors* – high psychological demands and low social support – were assessed using the 26 items of the French version of the Karasek Job Content Questionnaire (JCQ) (37). Scores were dichotomized using the median values of the French national SUMER study to classify exposed and unexposed workers (38).

### Statistical analysis

Analyses were conducted for the entire cohort, and a sex-stratified analysis was also performed to account for possible sex differences in occupational exposures (39, 40).

### Assessment of risk factors and population-attributable fraction (PAF) estimate in the COSALI cohort

Using a Cox multivariable regression model with constant follow-up time for each subject and robust variance (41), relative risks (RR) and their 95% confidence intervals (CI) were estimated for incident UEMSD occupational risk factors after adjustment for personal risk factors (age, sex, and BMI) in the COSALI cohort.

To quantify the proportion of UEMSD incident cases attributable to each risk factor, PAF were estimated for each risk factor in the multivariable model in addition to a combined PAF of all occupational factors. Point estimates and 95% CI of the PAF were calculated using the method described by Spiegelman et al (42). The PAF estimate accounted for the prevalence of the exposure and RR of UEMSD risk associated with that exposure (42):


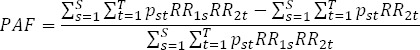


where *t* denotes a stratum of unique combinations of levels of all background risk factors which are not under study, *t=1;…; T*, and *RR_2t_* is the relative risk in combination *t* relative to the lowest risk level, where *RR_(2,t)_=1*. *s* indicates an index exposure group defined by each of the unique combinations of the levels of the index risk factors, that is, those risk factors to which the PAFapplies, *s=1;…; S*, and *RR_1s_* is the relative risk corresponding to combinations relative to the lowest risk combination *RR_1,1_=1*. The joint prevalence of exposure group *s* and stratum *t* is denoted by *p_st_*, and 
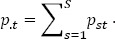
.

The calculation of PAF is recommended for multifactorial diseases when some risk factors are unmodifiable or not expected to change after intervention (43). To facilitate the comprehension and interpretation of the PAF estimate, the lower limit of its 95% CI was set to zero when this lower limit was negative.

### Estimated number of incident UEMSD attributable to occupational risk factors

To estimate the number of incident cases of UEMSD attributable to occupational risk factors in the PdL region, the calculation procedure was implemented in two steps. First, the study sample was weighted to provide estimates of incident UEMSD cases which were representative of the PdL working population, using data from the 2007 population census of the PdL region [conducted by the French National Institute of Statistics and Economic Studies (INSEE)]. A calibration on margins, proposed by Deville et al (44, 45), was used to take the characteristics of the PdL working population into account. The new weights were calculated using the following auxiliary variables (also called calibration variables): age, sex, occupational class and economic sector. These auxiliary variables were measured in both the COSALI cohort and the 2007 French population census, ie, their population distribution was known, and were correlated with the variable of interest, ie, incident UEMSD (according to Spearman’s correlation test). The “*linear”* calibration method was used to calculate the new weights from the “*Calmar*” macro (*calibration on margins*) developed by Sautory (46). With the calibration method, weights are assigned to all survey respondents in order to make the sample as representative as possible of the (inference) population. Over-represented groups then had a small weight and under-represented groups a large weight. The weighted sample (ie, with the new weights) is more representative of the working population of the PdL region, resulting in estimates with a lower bias than those that are unweighted. Furthermore, through the calibration method, potential improvements in the accuracy of the estimates can be expected (47).

At the second step, the estimated number of incident cases of UEMSD (and the variation range) attributable to risk factor was obtained by multiplying the PAF (and the 95% CI) by the projected number of incident UEMSD in the PdL region in 2007.

All statistical analyses were performed using the SAS software, version 9.4 (SAS Institute Inc, Cary, NC, USA).

## Results

### Study sample characteristics

Of the 1385 eligible participants with a standardized clinical examination at follow-up, a total of 1246 participants (734 (59%) men and 512 (41%) women) with a mean age of 38.2 (standard deviation 8.7) years at baseline were included in current analyses (figure 1). A comparison of characteristics and working conditions at baseline between the eligible participants included in the analyses and those excluded is provided in an additional file [see supplementary material, www.sjweh.fi/show_abstract.php?abstract_id=3911]. Excluded participants did not differ in terms of BMI, diabetes mellitus and rheumatoid arthritis, but were significantly older than those included in analyses (P<0.001). They were more likely to: be women (P=0.036) and lower-grade white-collar workers (P<0.001), work in trade and services sectors (P<0.001), be temporarily employed (P=0.006) or have a higher seniority level in their current job (P= 0.041). No difference was observed in working conditions under study. However, borderline differences exist for perceived physical exertion, repetitiveness of task, and use of vibrating tools.

### Incident UEMSD diagnosed at follow-up

At least one of the six UEMSD was diagnosed at follow-up in 139 workers free from UEMSD at baseline (74 men and 65 women) corresponding to a projected number of 129 320 new UEMSD cases in the PdL region in 2007 (table 1). The incidence proportion of UEMSD observed in the PdL region did not significantly differ between sexes (10.3% for men versus 12.4% for women; P=0.287). The most common diagnoses at follow-up were rotator cuff syndrome (incidence proportion 6.5%), lateral epicondylar tendinopathy (incidence proportion 2.2%) and CTS (incidence proportion 2.0%). The estimate of the projected number of workers in the PdL with two or more UEMSD at follow-up was 19 404 (1.7%) workers.

**Table 1:** Distribution of the six upper-extremity musculoskeletal disorders (UEMSD) among the study population and its projection.

	Study sample							Projection of the study sample at the level of the PdL region						
	
	Overall (N=1246)		Men (N=734)		Women (N=512)		P-value ^a^	Overall (N=1141 324) ^b^		Men (N=582 950) ^b^		Women (N=558 373) ^b^		P-value ^c^
	N	%	N	%	N	%		N	%	N	%	N	%	
Rotator cuff syndrome	78	6.3	41	5.6	37	7.2	0.242	73 858	6.5	32 827	5.6	41 032	7.3	0.259
Lateral epicondylar tendinopathy	28	2.3	22	3.0	6	1.2	0.032	24 767	2.2	18 117	3.1	6650	1.2	0.033
Carpal tunnel syndrome	24	1.9	7	1.0	17	3.3	0.003	22 456	2.0	7228	1.2	15 228	2.7	0.084
Ulnar tunnel syndrome	12	1.0	7	1.0	5	1.0	1.000 ^d^	12 022	1.1	7796	1.3	4227	0.8	0.332
De Quervain tenosynovitis	10	0.8	4	0.6	6	1.2	0.334 ^d^	7878	0.7	2159	0.4	5719	1.0	0.138
Flexor-extensor peritendinitis or tenosynovitis of the forearm-wrist region	9	0.7	5	0.7	4	0.8	1.000 ^d^	9399	0.8	3988	0.7	5410	1.0	0.625
≥1 of 6 UEMSD	139	11.2	74	10.1	65	12.7	0.149	129 320	11.3	60 133	10.3	69 187	12.4	0.287
≥2 of 6 UEMSD	20	1.6	11	1.5	9	1.8	0.720	19 404	1.7	10 921	1.9	8483	1.5	0.652

a P-value of Chi-square test;

b Weighted.

c P-value of the Rao-Scott Chi-square test for weighted samples.

d Fisher’s exact test.

### Incident UEMSD risk factors

The RR for incident UEMSD associated with occupational risk factors in the multivariable model after adjustment for personal risk factors are shown in table 2. The following occupational exposures were positively associated with incident UEMSD: high perceived physical exertion (RR 1.52, 95% CI 1.06−2.17), working with arms above shoulder level (RR 1.57, 95% CI 1.04−2.39) and low social support at work (RR 1.41, 95% CI 1.03−1.92).

**Table 2:** Multivariable models for risk factors of incident upper-extremity musculoskeletal disorders (UEMSD) in the COSALI cohort. [RR=relative risk; 95% CI=95% confidence interval]

	Overall study sample (N=1246; incident UEMSD=139)			Men (N=734; incident UEMSD=74)			Women (N=512; incident UEMSD=65)		
		
	N (%)	RR (95% CI)	P-value ^a^	N (%)	RR (95% CI)	P-value ^a^	N (%)	RR (95% CI)	P-value ^a^
Biomechanical factors ^b^									
High perceived physical exertion (≥13) ^c^	571 (45.8)	1.52 (1.06−2.17)	0.022	365 (49.7)	2.38 (1.41−4.04)	0.001	206 (40.2)	0.74 (0.41−1.33)	0.319
High repetitiveness of tasks (>4 hrs/day)	267 (21.4)	1.15 (0.81−1.64)	0.421				128 (25.0)	1.33 (0.86−2.08)	0.201
Repeated/sustained posture with arms above shoulder level (≥2 hrs/day)	126 (10.1)	1.57 (1.04−2.39)	0.033	76 (10.4)	1.28 (0.71−2.33)	0.412	50 (9.8)	1.70 (0.97−2.98)	0.066
Repeated/sustained posture with shoulder abduction ^d^	373 (29.9)	1.26 (0.88−1.81)	0.201				126 (24.6)	1.75 (1.05−2.93)	0.032
Repeated/sustained elbow movements (flexion/extension) (≥2 hrs/day)	355 (28.5)	1.00 (0.69−1.46)	0.994	213 (29.0)	1.26 (0.79−2.00)	0.327			
Wrist twisting movements (≥2 hrs/day)	386 (31.0)	0.99 (0.67−1.46)	0.970				148 (28.9)	1.41 (0.82−2.41)	0.214
Psychosocial factors ^e^									
Low social support	444 (35.6)	1.41 (1.03−1.92)	0.032	279 (38.0)	1.42 (0.92−2.17)	0.109	165 (32.2)	1.36 (0.85−2.17)	0.196
High psychological demands				348 (47.4)	1.29 (0.84−1.99)	0.244			
Personal factors									
Female sex	512 (41.1)	1.33 (0.97−1.81)	0.075						
Age: 35–44 years (ref: <35 years)	451 (36.2)	1.54 (1.03−2.29)	0.034	268 (36.5)	1.80 (1.02−3.16)	0.041	183 (35.7)	1.37 (0.77−2.42)	0.286
Age: ≥45 years (ref: <35 years)	335 (26.9)	2.13 (1.44−3.16)	<0.001	186 (25.3)	2.77 (1.59−4.83)	<0.001	149 (29.1)	1.60 (0.92−2.78)	0.098
Overweight/obesity ^f^							124 (24.2)	1.70 (1.07−2.72)	0.025

a P-value of Wald test.

b Binary variables assessed using exposure criteria from the European consensus criteria to diagnose work-related UEMSD (32).

c Assessed using the Borg RPE scale (35).

d Workers who responded “rarely (<2 hours/day)”, “often (2–4 hours/day)” or “always (≥4 hours/day)” were defined as being at risk of this posture (30).

e Binary variables assessed using assessed using the French JCQ (38).

f Binary variable assessed using the World Health Organization criteria (33).

Concerning personal factors, age was associated with the incident UEMSD while the RR for female sex was at the limit of statistical significance.

### PAF and estimated number of incident UEMSD attributable to risk factors

PAF associated with the incidence of UEMSD in the multivariable model were 20.4% (95% CI -1.1−40.1) for high physical exertion (Borg RPE scale ≥13), 6.6% (-3.5−16.4) for working with arms above shoulder level (≥2 hours/day), and 12.9% (0.3−25.1) for low social support (table 3). Of the projected estimate of 129 320 incident UEMSD cases in PdL in 2007, an estimated 26 381 (variation range: 0−51 857) new UEMSD cases were attributable to high physical exertion, 16 682 (388−32 459) to low social support, and 8535 (0−21 208) new cases to working with arms above shoulder level. A high number of incident UEMSD [10 863 cases (0−30 778)] could be attributed to working with shoulder abduction despite the associated RR failing to reach the 5% statistical significance level. The projected number of incident UEMSD attributable to all occupational factors in the multivariable model was estimated at 53 021 (0−98 671) cases, representing 41.0% of all new UEMSD in the PdL region.

**Table 3:** Population-attributable fraction (PAF) and estimated number (EN) of incident upper-extremity musculoskeletal disorders (UEMSD) attributable to risk factors. PAF was adjusted for all factors in the model and calculated using the lowest risk group for each factor as the reference group, with all other factors remaining unchanged. EN was calculated by multiplying the PAF by the projected number of incident UEMSD cases in the Pays de la Loire region in 2007.

	Overall population				Men				Women			
		
	PAF ^a^	95% CI	EN	EN variation range	PAF^a^	95% CI	EN	EN variation range	PAF ^a^	95% CI	EN	EN variation range
Biomechanical factors									
High perceived physical exertion (≥13)^a, b^	20.4	-1.1−40.1	26 381	0−51 857	41.6	14.2−63.1	25 015	8539−37 944				
High repetitiveness of tasks (>4 hrs/day)	3.7	-6.7−14.0	4785	0−18 105					8.9	-8.6−25.8	6158	0−17 850
Repeated/sustained posture with arms above shoulder level (≥2 hrs/day)	6.6	-3.5−16.4	8535	0−21 208	3.9	-7.2−14.8	2345	0−8900	7.6	-5.7−20.6	5258	0−14 253
Repeated/sustained posture with shoulder abduction	8.4	-7.4−23.8	10 863	0−30 778					17.8	-7.5−41.0	12 315	0−28 367
Elbow flexion/extension movements (≥2 hrs/day)	0.1	-12.6−12.7	129	0−16 424	7.8	-9.2−24.4	4690	0−14 672				
Wrist twisting movements (≥2 hrs/day) ^b^									12.4	-13.0−36.4	8579	0−25 184
Psychosocial factors								
Low social support	12.9	0.3−25.1	16 682	388−32 459	14.3	-4.3−32.0	8599	0−19 243	10.6	-6.7−27.2	7334	0−18 819
High psychological demands					11.9	-8.3−31.2	7156	0−18 761				
Personal factors								
Female sex	11.5	-1.0−23.6	14 872	0−30 520								
Age: 35–44 years	12.6	2.7−22.3	16 294	3492−28 838	16.8	2.7−30.3	10 102	1624−18 220	9.1	-5.1−22.8	6296	0−15 775
Age: ≥45 years	19.9	11.6−27.8	25 735	15 001−35 951	24.2	14.4−33.5	14 552	8659−20 145	13.8	-1.9−28.9	9548	0−19 995
Overweight/obesity									15.3	-1.7−31.4	10 586	0−21 725
All occupational factors	41.0	-13.0−76.3	53 021	0−98 671	59.7	4.4−87.0	35 899	2646−52 316	42.5	-20.8−80.7	29 411	0−55 834

a Assessed using the Borg RPE scale (35).

b Relative risk <1 and the PAF was not calculated.

### Sex-stratified analyses

Results from sex-stratified analyses suggest that the observed relationship between incident UEMSD and high physical exertion or low social support were primarily observed among men. The relationship observed between incident UEMSD and sustained or repetitive shoulder abduction or working with arms above shoulder level were primarily observed among women. Thus, the association of high physical exertion with incident UEMSD was only statistically significant (RR 2.38, 95% CI 1.41−4.04)] among men, while the association with low social support approached statistical significance (RR 1.42, 95% CI 0.92−2.17). Occupational exposure with shoulder abduction was only found to be positively associated with incident UEMSD (RR 1.75, 95% CI 1.05−2.93) among women, and the RR associated with working with arms above shoulder level approached statistical significance (RR 1.70, 95% CI 0.97−2.98) (table 2).

Of the projected total estimate of 60 133 UEMSD incident cases among male workers in the PdL region in 2007, 25 015 were attributable to high physical exertion, representing 41.6% of all new cases, while 8599 (14.3%) could be attributed to low social support (table 3). Similarly, of the projected 69 187 new UEMSD cases among women estimated in 2007, 12 315 cases (17.8% of all new UEMSD) were attributable to working with shoulder abduction while 5258 cases (7.6%) could be attributed to working with arms above shoulder level. In addition, the PAF among women for being overweight/obese (a potentially modifiable factor) was 15.3% corresponding to 10 586 new UEMSD cases in the PdL region in 2007.

The PAF attributable to all occupational factors was estimated to be 59.7% among men and 42.5% among women, corresponding to 35 899 and 29 411 projected incident cases of UEMSD in the PdL region, respectively.

## Discussion

### Main findings

This study has estimated the number of incident cases of UEMSD attributable to occupational exposure factors in the working population of the French PdL region in 2007.

Considering occupational risk factors for incident UEMSD, our results showed that an estimated 26 381 incident cases, representing 20.4% of all new projected UEMSD cases in the PdL region in 2007, were attributable to high physical exertion, 8535 incident cases (6.6%) to working with arms above shoulder level, and 16 682 incident cases (12.9%) to low social support from coworkers and supervisors. Furthermore, a significant number of new UEMSD cases (N=10 863) could be attributed to working with shoulder abduction despite the associated RR did not reach the 5% statistical significance level.

### Comparison with previous literature

To our knowledge, this is the first cohort study estimating the number of potential cases of UEMSD attributable to occupational risk factors in an entire working population.

The main occupational factor likely to lead to the highest number of incident cases of UEMSD was high physical exertion, associated with 26 381 cases (about 20.4% of incident UEMSD in the PdL working population). Approximately one in five incident UEMSD could theoretically be prevented by reducing exposure to physical exertion in the workplace. Previous studies carried out in Italy and the Netherlands (22, 23) reported that 28% of CTS cases and 25% of lateral epicondylar tendinopathy cases respectively, could be attributable to high physical exertion. Moreover, a recent narrative review showed that forceful exertion was a significant risk factor for all UEMSD (48). Meta-analyses have also revealed a significant relationship between shoulder disorders and hand force exertion, but with moderate evidence (11), and between CTS and force (10). In addition, a summary study based on three longitudinal MSD studies provided strong evidence for a relationship between lateral epicondylalgia and occupational exposure to high hand force (49).

Our study indicated the important contribution of awkward shoulder postures with a projected estimate of 8535 (6.6%) and 10 863 (8.4%) incident UEMSD attributable to working with arms above shoulder level and working with shoulder abduction respectively. This result is consistent with recent PAF estimates (15% for lateral epicondylar tendinopathy and 9% for shoulder disorders) associated with awkward postures in the working population (22) and a recent meta-analysis showing moderate evidence of a positive association between shoulder disorders and exposure to arm-hand elevation (11).

The present study estimated the projected number of incident UEMSD related to low social support at 16 682, representing 12.9% of all incident cases. Our PAF estimates are in line with the findings from the 2001 US National Research Council extensive review (27), which concluded that improving low social support of coworkers and supervisors in exposed workers could potentially reduce the risk for UEMSD by 28–52%. A multitude of psychosocial factors in the workplace, including poor social support, activate psychosocial stress. Stress then appears to initiate a sequence of physiological reactions, including biochemical reactions, which in the short term may increase muscle tension and, in the long term, may increase the risk of MSD (50). Therefore, an improvement in social support from superiors and colleagues may contribute to the reduction of this risk. Moreover, workers with low social support may be exposed to higher levels of biomechanical risk factors (51). Conversely, high social support may facilitate the cooperation between coworkers in performing strenuous manual tasks to minimize biomechanical exposure (52). In a previous meta-analysis, exposure to low social support in the workplace was positively associated with the onset of UEMSD (9). A systematic literature review showed that low social support at work may result in an increased occurrence of specific disorders at the elbow (12). Another systematic review by Kraatz et al (53) showed strong evidence for adverse effects of low social support on the onset of shoulder disorders. A meta-analysis of Lang et al (54) found positive associations between psychosocial work stressors, including low social support at work, and shoulder symptoms and upper-extremity symptoms, while another found low-quality evidence of no association for social support (11). However, this finding has been inconsistent with some previous studies. A prospective study found no associations between social support and incident UEMSD (lateral epicondylitis, rotator cuff tendinitis, CTS, tendinitis of forearm–wrist extensors and flexors) (55). Recently, a review reported limited evidence for a positive association between psychosocial factors including low social support and CTS in the workplace (56).

Concerning personal risk factors, sex and age are not modifiable factors. Among the potentially personal modifiable risk factors, the present study suggests that (in women) an important number of projected incident UEMSD could be attributed to high BMI (15.3% of all projected new cases) in the PdL female working population. This result is in line with a prospective cohort study of Italian workers reporting that about 30% of CTS cases may be attributable to being overweight/obese (23). These differences may reflect the gender division of work where men are more often exposed to jobs requiring high physical work load and forceful exertion (eg, in the construction sector) and women more often exposed to highly repetitive tasks with lower force exertion (eg, in assembly line work) (39, 40). Moreover, highly physically demanding jobs (eg, manual handling of heavy loads) require mutual help and social support from coworkers to collectively cope with job tasks and minimize biomechanical exposure (52).

### Strengths and limitations

The present study has some limitations. Approximately 57% of workers included at baseline did not have a follow-up clinical examination. Within this participant group, 58% were no longer being monitored by any OP of the network because they had left their baseline jobs without informing their OP. In some cases, their OP refused to participate in the follow-up period. Moreover, the follow-up period coincided with the major economic crisis in the PdL region in 2008–2009, during which the regional salaried workforce declined by 3.4% (33.7% in temporary employment agencies) (57). The lowest participation rate in this study was among young or temporary workers or those with a short length of service at baseline (29). According to a study on the effects of drop out in a longitudinal study of MSD (58), the differences between the participants and the drop out subjects had a very modest influence on the RR for effects of occupational exposures. We therefore believe that there was no major selection bias associated with the quality of the follow-up.

Another limitation is the exposure assessment, which was based only on workers self-reporting. In spite of that, the use of standardized and validated questions may have ensured better quality of the self-reported exposure measures. Non-differential misclassification of exposures may have occurred due to workers’ inability to precisely recall or describe their current work exposures among workers without symptoms. Nevertheless, due to the prospective design of the study, exposure information gathered prior to UEMSD diagnosis resulted in low risk of differential recall bias. To the extent that the risk of UEMSD is increased by cumulative physical exposures, our analyses may have underestimated the true contribution of work exposures to the incidence of UEMSD in our study population. This may be particularly the case for rotator cuff syndrome, since studies of work-related risk factors for shoulder pain have identified the length of time employed as a risk factor (59). The single and short window of follow-up in this study after a long follow-up period is another potential limitation. Workers may have had a UEMSD in the period between the first and second clinical examinations, but recover and do not have the UEMSD at follow-up. This may have resulted in an underestimation of the number of cases diagnosed.

The computation of the combined PAF assumes independence and the absence of interaction between individual risk factors. However, there may be an interaction between certain occupational risk factors. In such cases, the calculation of the combined PAF may lead to its over- or underestimation. Nevertheless, none of the interactions between occupational exposures explored previously was statistically significant (29). It should also be noted that the choice of thresholds used to define exposure levels can have an effect on PAF estimates (60). However, to avoid bias, we chose exposure definitions as close as possible to public health recommendations and those recommended in the scientific literature. The concept of PAF supposes a causal relationship between exposure and UEMSD (19). Moreover, a strong association between a risk factor and UEMSD, ie, a high RR, may correspond to a low or high PAF depending on the prevalence of exposure. This leads to very different public health consequences as the prevalence of exposure can vary considerably within populations that are separated in time and space (61). Thus, we assume that a reduction in occupational exposure at the working population level would lead to a reduction in the incidence of WRUEMSD and PAF estimates should therefore be interpreted with caution. Finally, it is possible that the 95% CI of the PAF includes the null value, despite the significance of the RR due to the use of nonlinear transformations to compute the 95% CI of the PAF (62). Even so, zero should be close to the 95% CI.

The use of a prospective cohort including a representative sample of the working population at baseline is a major strength of the present study (31). Secondly, outcomes were clinically assessed by trained OP using standardized procedures (31, 32). In addition, this study strictly applied the definitions of exposures proposed by the European consensus criteria document (32), except for the measure of exposure to forceful exertion which was assessed according to the rating of perceived exertion (35) and the INRS cut-offs (36).

Another strength is the formula used to estimate the PAF from multivariable regression models, allowing a non-biased computation of PAF estimates adjusted for covariates (19). Lastly, sophisticated weighting adjustment methods (44–46) for enhancing estimate accuracy were used to extrapolate the number of cases observed in the study sample to the whole working population. Furthermore, the “*linear”* calibration method used to calculate the new weights was the one that gave the lowest variance and range of weight ratios (new weights / initial weights). Indeed, it was chosen by considering the following criteria: lowest dispersion, smallest extent and general appearance of the distribution of the new weight distribution; the other calibration methods give calibrated estimators with the same asymptotic accuracy (44, 46).

Finally, estimating the number of incident cases of UEMSD in the working population of the PdL region is useful for comparing the population-level impacts of various risk factors on the incidence of UEMSD. Furthermore, these estimates provide additional input for the implementation of prevention programs that target and prioritize the modifiable risk factors with the greatest impact for more effective interventions to reduce the medical, economic and social impact of UEMSD in the workplace.

### Concluding remarks

Following adjustment for personal factors, we have been able to estimate the proportion and projected number of new UEMSD cases attributable to occupational risk factors in the working population of the French PdL region. According to our findings, an important proportion and a large number of incident UEMSD in the workplace in the PdL region could potentially be prevented by reducing occupational exposures such as physical exertion, working with shoulder abduction, and improving social support from coworkers and supervisors. These findings highlight the magnitude of potentially modifiable and preventable occupational exposures in the incidence of UEMSD in the workplace.

## Supplementary material

Supplementary material
