# Supplementary material for: Upper-extremity musculoskeletal disorders: how many cases can be prevented? Estimates from the COSALI cohort
Source: Scand J Work Environ Health. 2020 Oct 30;46(6):618–29. doi: 10.5271/sjweh.3911 (PMC7737791; doi:10.5271/sjweh.3911)
Supplement: Supplementary material [file SJWEH-46-618-S001.pdf]

Upper-extremity musculoskeletal disorders: how many cases can be prevented? Estimates from the COSALI cohort [1]

by Aboubakari Nambiema, MSc, MPH,[2] Julie Bodin, PhD, Natacha Fouquet, PhD, Sandrine Bertrais, PhD, Susan Stock, MD, MSc, FRCPC, Agnès Aublet-Cuvelier, MD, Alexis Descatha, MD, PhD, Bradley Evanoff, MD, MPH, Yves Roquelaure, MD, PhD

1. *Supplementary material*
2. *Correspondence to: Aboubakari Nambiema, MSc, MPH, Irset - Inserm UMR 1085 - Equipe Ester, Faculté de santé - Département Médecine, 28 Rue Roger Amsler, 49100 Angers, France. [E-mail: aboubakari.nambiema@univ-angers.fr] ORCID iD: orcid.org/0000-0002-4258-3764*

Table S1: A comparison of characteristics and working conditions at baseline between the eligible participants included in the analyses and those excluded

	**Included participants (n = 1246)**		**Excluded participants (n = 139)**		**P**
	**n**	**%**	**n**	**%**	
**Baseline characteristics**					
Sex					0.036
Men	734	58.9	69	49.6	
Women	512	41.1	70	50.4	
Age (years)					<0.001
<35	460	36.9	35	25.2	
35-44	451	36.2	44	31.7	
≥45	335	26.9	60	43.2	
Overweight/obesity	436	35.2	44	33.1	0.628
Diabetes mellitus	13	1.0	2	1.4	0.656*
Rheumatoid arthritis	20	1.6	2	1.4	1.000*
Seniority in current job (years)					0.041
<1	107	8.7	14	10.3	
1-2	196	15.9	17	12.5	
3-10	463	37.5	38	27.9	
>10	470	38.0	67	49.3	
Occupational class					<0.001
Craftsmen, salesmen and managers	0	0.0	3	2.2	
Professionals	95	7.6	6	4.4	
Technicians, associate professionals	300	24.1	34	24.8	
Low-grade white-collar workers	345	27.7	50	36.5	
Blue-collar workers	506	40.6	44	32.1	
Temporary employment	70	5.6	16	8.6	0.006
Economic sector					<0.001
Agriculture	0	0.0	13	9.4	
Industry	477	38.3	26	18.8	
Construction	63	5.1	9	6.5	
Trade and services	706	56.7	90	65.2	
**Working conditions at baseline**					

**Biomechanical factors**					
At least one of the six UEMSD#	139	11.2	13	10.5	0.820
High perceived physical exertion (Borg RPE scale ≥13)	571	45.8	72	54.6	0.056
High repetitiveness of tasks (>4 hours/day)	267	21.4	36	28.8	0.058
Use of vibrating tools (≥2 hours/day)	158	12.7	10	7.5	0.079
Repeated/sustained posture with arms above shoulder level (≥2 hours/day)	126	10.1	18	13.5	0.220
Repeated/sustained posture with shoulder abduction	373	29.9	13	44.8	0.084
Repeated/sustained elbow movements (flexion/extension) (≥2 hours/day)	355	28.5	37	27.6	0.830
Pronation and supination movements (≥2 hours/day)	156	12.5	20	15.2	0.389
Wrist twisting movements (≥2 hours/day)	386	31.0	40	31.7	0.859
Use of the pinch grip (≥4 hours/day)	87	7.0	7	5.3	0.455
**Psychosocial factors**					
Low social support	444	35.6	40	36.4	0.878
Low decision latitude	626	50.2	63	51.6	0.372
High psychological demands	609	48.9	58	45.3	0.442

P: p-value of independent Khi-2 test; *Fisher's exact test; #UEMSD: upper-extremity musculoskeletal disorders
